# Diet Quality, Nutritional Adequacy, and Sociodemographic Characteristics of Mobile Food Pantry Users in Northeastern Connecticut

**DOI:** 10.3390/nu13041099

**Published:** 2021-03-27

**Authors:** Dalia Marmash, Kyungho Ha, Junichi R. Sakaki, Isabella Gorski, Brazil Rule, Jaime Foster, Michael Puglisi, Ock K. Chun

**Affiliations:** 1 Department of Nutritional Sciences, University of Connecticut, Storrs, CT 06269, USA; dalia.marmash@uconn.edu (D.M.); junichi.sakaki@uconn.edu (J.R.S.); isabella.gorski@uconn.edu (I.G.); brazil.rule@uconn.edu (B.R.); michael.puglisi@uconn.edu (M.P.); 2 Department of Food Science and Nutrition, Jeju National University, Jeju 63243, Korea; kyungho.ha@jejunu.ac.kr; 3 Connecticut Food Bank, Wallingford, CT 06492, USA; JFoster@ctfoodbank.org

**Keywords:** sociodemographic, diet quality, nutrient adequacy, food security, mobile food pantry, low-income

## Abstract

Poor diet quality among low-income populations is a major contributing factor to their poor health and wellbeing, and thus is a focus of many government aid programs. Mobile food pantries are an increasingly popular method of emergency food assistance, targeting the communities most affected by food insecurity; however, little is known about the dietary characteristics of mobile food pantry users. This study aims to characterize the diet quality and nutrient adequacy level and examine its association with sociodemographic characteristics among mobile food pantry users in Windham County, Connecticut. Surveys to assess food insecurity, diet composition, and sociodemographic characteristics were administered to 83 adult food pantry users. Participants (*n* = 40) completed a three-day dietary record for analysis of diet quality, and were found to have inadequate intakes of fruits, vegetables, whole grains and dairy, as well as some related micronutrients. At least 30% of participants had intakes below the Estimated Average Requirement (EAR) for vitamins A, C, E, calcium, zinc, magnesium, and folate. Intakes of added sugar, sugar sweetened beverages, and saturated fat were also above recommendations according to the United States Dietary Guidelines. Certain sociodemographic factors affected diet quality among this sample. For example, being male was associated with increased sugar-sweetened beverage and added sugar intake. This characterization of mobile pantry users will serve as a reference for developing nutrition education and determining the effectiveness of future interventions.

## 1. Introduction

Food insecurity is a startlingly prevalent challenge faced by millions of Americans, leading to numerous dietary disparities. Food insecurity—defined as not having the right types, or amount, of food to feed all individuals in the household—disproportionately affects minorities and those living below the federal poverty line [1]. Even though Connecticut is one of the wealthiest states in the country, it has over 400,000 residents struggling with hunger [2]. Furthermore, the wealth distribution in Connecticut has led to pockets of low-income residents throughout the state. Windham County, in the Northeastern corner of the state, has the lowest county-wide median income in Connecticut [3]. The food insecurity rate is 16.4% and the poverty rate is 25.4%, higher than the national and state average [3].

There are numerous resources available to these low-income, food insecure individuals. The Connecticut Food Bank has a network of food pantries, soup kitchens, and other emergency food assistance available to all residents [4]. Mobile food pantries are an increasingly popular method of distribution, allowing for the delivery of perishable foods to those in need [4,5]. Bi-weekly distribution of fruits, vegetables, meats, dairy, and pantry staples supplements other assistance residents have access to [4]. Food is distributed at several convenient community locations—allowing for those without access to reliable transportation, such as elderly, extremely low income, or disabled individuals, the opportunity to receive assistance. This differs from traditional food pantry distribution, which often consists of non-perishable, shelf-stable items. There is a large body of research assessing the diet quality of traditional food pantry users, but very few studies have been conducted with mobile pantry users.

Food pantry users have diets that often are not in alignment with the US Dietary Guidelines [6,7,8,9]. More specifically, intakes of fruits, vegetables and dairy are low in food pantry users [7,8,9,10,11]. There are also low intakes of micronutrients related to these food groups, such as calcium, vitamin D, vitamin A, and magnesium [9,12]. In general, diet quality and nutritional adequacy are inversely associated with income [13], largely due to the high cost of purchasing and storing nutritious foods [13,14], as well as their limited availability in many low-income areas [15]. Furthermore, income and education levels are inversely associated with energy density and positively associated with diet quality [6]. More specifically, those with higher incomes and education levels spend more per calorie than those of lower socioeconomic status, suggesting a close relationship between diet quality and diet cost [6].

Previous literature has focused on traditional food pantry distribution and diet quality [7,8,10,12,16,17,18]. Mobile food pantries operate differently than traditional pantries—increased distribution of fresh foods along with increased accessibility to community members in need [5]. Furthermore, interventions targeting the food quality of traditional food pantries have seen increases in diet quality with increased distribution of fruits, vegetables, and dairy products [12,19]. This suggests that data from traditional pantries might be less applicable to mobile pantry users since fresh foods are more widely available at mobile pantries.

The current study aims to characterize mobile food pantry users in Windham County—determining key nutrition and public health areas of concern. This population is unique in its largely Hispanic or Latinx make-up. Many previous characterizations of food pantry users are in largely Non-Hispanic White or Non-Hispanic Black populations [7,8,16]. Evidence has suggested the possibility of racial differences in diet composition and thus diet quality, making previous research even less generalizable to the population of this study [20]. This assessment will give researchers insight into the most prevalent concerns, and will provide insight into the diet quality of mobile food pantry users specifically.

## 2. Materials and Methods

### 2.1. Study Design and Participants

Participants were recruited from two mobile food pantry sites in Windham County, CT. The first, Windham Heights, is a low-income housing complex located in Willimantic. The second, The First Congregational Church, is located in downtown Willimantic. Willimantic was chosen due to its high prevalence of food insecurity and poverty [3].

Participants had to be at least 19 years of age and had to have visited a food pantry at least once prior to study commencement to be included. Those who met the inclusion criteria were given a copy of the consent form, which was clarified by research staff. Consent forms were signed prior to study commencement. The study protocol was approved by the University of Connecticut Institutional Review Board (H19-206).

Research assistants administered three interviews. The first was study-specific, aimed at obtaining sociodemographic information and frequency of food pantry visits. Next, the United States Department of Agriculture (USDA) Food Security Questionnaire and the National Cancer Institute (NCI) Dietary Screener Questionnaire (DSQ) were administered [21,22]. Participants were asked to complete a 3-day dietary record on three non-consecutive days (two weekdays and one weekend day) and trained by research staff on recording all food and nutritive beverages consumed. They were then asked to return the food record in provided pre-stamped envelopes. The participants were compensated throughout the study process. Following the first day of interviews, a USD 20 Walmart gift card was awarded, and an addition USD 10 was given to those who returned their completed 3-day food records. A total of 83 participants were recruited from the two sites. Forty participants sent in their 3-day food record, completing the study.

### 2.2. Sociodemographic Characteristics

Sociodemographic data including race, education, employment, income, marital status and assistance program use were collected. Poverty income ratio (PIR) was calculated by dividing the household income by the poverty threshold. Since income was reported as a range, a median value was used for this calculation. The poverty threshold is determined by family size, the number of older adults up to 2, and the number of children under 18 years old [23]. Participants were then divided into tertiles according to PIR. In addition, participants were divided into three categories based on their household income in comparison to the poverty thresholds: above poverty (greater than the thresholds), poverty (less than or equal to the thresholds), or extreme poverty (less than 50% of the thresholds).

### 2.3. Food Security

Food security was determined using the USDA Household Food Security survey. A series of 18 questions about food quality and availability were administered. Distinction of food security class followed methods published by the USDA [21]. If participants responded “often” or “sometimes”, a score of a 1 was given. If the participant responded “never true” or “I don’t know”, a 0 was given. Total scores were calculated, with higher scores corresponding to more severe food insecurity. A total score of 2–4 was categorized as low food security and a score of 5 or higher was considered to be very low food security.

### 2.4. Diet Quality and Nutrient Adequacy

The NCI DSQ was used to estimate the daily intake of food groups of the 83 participants. This 26-item questionnaire includes questions about the frequency of consumption of a variety of foods over the past 30 days [24]. These responses are then translated to predicted daily intakes of 9 food groups including whole grains, added sugars, dairy, fruits, vegetables, and sugar-sweetened beverages. Predicted intakes were calculated using scoring algorithms developed by the NCI, which have been validated for the estimation of dietary intake [25]. DSQ data allowed for determination of dietary patterns over the last month—giving a more general idea of diet quality among participants. The questionnaire was completed with research staff to ensure understanding and accuracy of responses.

Diet quality was also assessed using the 3-day food record data from the 40 participants, which was input into Nutrition Data System for Research (NDSR) 2019 software (University of Minnesota, Minneapolis, MN, USA). Daily nutrient intake along with HEI-2015 scores were calculated. HEI-2015 consists of 13 components scored based on adherence to the 2015–2020 Dietary Guidelines for Americans [26]. Total scores range from 0–100, with a higher score indicating greater adherence to the Dietary Guidelines. Nutrient adequacy was determined by calculating the number of participants who did not meet the acceptable macronutrient distribution range (AMDR), estimated average requirement (EAR), or the adequate intake (AI) [27]. Using 3-day food records allowed for a snapshot of dietary intake following the mobile food pantry visit [28]. Participants were asked to complete the record on non-consecutive days, two weekdays and one weekend, to more accurately estimate intake [29]. Participant misreporting was assessed using acceptable energy ranges as described by Willett [29]. This protocol has been used to assess plausibility of reported dietary intake [30,31]. In the current study, participants averaged 3-day energy intakes ranged from 582–3528 kcal/day for women and 1114–4110 kcal/day for men, which generally met allowable energy intake ranges used to examine plausibility of self-reported energy intake data (500–3500 kcal/day for women and 800–4000 kcal/day for men) [29].

### 2.5. Data Analysis

All statistical analyses were conducted using SAS software (version 9.4; SAS Institute, Cary, NC, USA). All values were presented as mean ± standard deviation (SD) or n (%). Differences in daily food group intakes by sociodemographic factors were examined using a general linear model and food group intake values were log-transformed to normalize distribution. Differences in nutrient inadequacy level by sociodemographic characteristics were examined using chi-square test or Fisher’s exact test. All *p* values reported were two-sided and *p* < 0.05 was considered statistically significant.

## 3. Results

### 3.1. Participant Sociodemographic Characteristics

Mobile food pantry users in Windham County, Connecticut were predominantly Latinx and Female (Table 1). Almost two-thirds of the participants were below the federal poverty line, with over two-thirds considered food insecure. Assistance program participation was also high, with 86% participating in at least one form of government assistance and 57% of participants visiting a food pantry in the last month.

Previous research similarly has a high proportion of female participants [7,16], but the largely Latinx make-up of the sample is unique. As expected, food security and income are low compared to state averages—as Windham county is the poorest in Connecticut [3].

### 3.2. Diet Quality of Mobile Food Pantry Users

Recommended food group intake based on the USDA Dietary Guidelines for Americans is shown in Table 2 [26], and are based on the average 2000 calorie diet.

Table 3 shows the associations between predicted food group intake and sociodemographic groups. Being male was associated with significantly higher intakes of added sugars, dairy, vegetables and legumes including French fries, and sugar sweetened beverages (SSB) than female participants (*p* < 0.05 for all). Intakes of added sugars, fruits, and SSB differed by age groups (*p* < 0.05 for all), with participants in the 19–30-year-old age groups having the highest consumption. Participating in 3 or more assistance programs was associated with higher fruit and SSB intake (*p* < 0.05 for all). There were no significant differences in intakes of food groups by race, education, employment, income, poverty status, last food pantry visit, or food security. 

**Table 3:** Predicted daily intakes of food groups by socioeconomic characteristics based on NCI Dietary Screener Questionnaire in mobile food pantry users in Windham County, CT (*n* = 83).

	Whole Grain (oz)	Added Sugars (tsp)	Dairy (Cup)	Fruits and Vegetables Including Legumes and French Fries (Cup)	Vegetables Including Legumes and Including French Fries (Cup)	Fruits and Vegetables Including Legumes and Excluding French Fries (Cup)	Vegetables Including Legumes and Excluding French Fries (Cup)	Fruits (Cup)	Sugar-Sweetened Beverages (tsp Sugar)
Sex									
Male (*n* = 16)	0.8 ± 0.4	19.6 ± 6.2 *	2.1 ± 0.6 *	3.1 ± 1.2	2.0 ± 0.7 *	3.0 ± 1.2	1.9 ± 0.7	1.1 ± 0.6	9.8 ± 4.9 *
Female (*n* = 67)	0.7 ± 0.4	15.5 ± 8.4	1.7 ± 0.9	2.8 ± 0.8	1.7 ± 0.5	2.7 ± 0.9	1.6 ± 0.5	1.1 ± 0.6	7.1 ± 7.1
Age (years)									
19–30 (*n* = 8)	0.8 ± 0.3	21.3 ± 15.3 *	2.2 ± 1.9	3.4 ± 1.2	1.8 ± 0.6	3.3 ± 1.3	1.7 ± 0.6	1.8 ± 1.0*	11.6 ± 11.6 *
31–44 (*n* = 13)	0.8 ± 0.6	21.1 ± 10.7	2.0 ± 0.8	2.5 ± 0.8	1.7 ± 0.5	2.4 ± 0.9	1.6 ± 0.5	1.0 ± 0.4	11.4 ± 10.3
45–64 (*n* = 41)	0.7 ± 0.4	14.9 ± 5.5	1.8 ± 0.7	2.9 ± 0.9	1.8 ± 0.5	2.8 ± 0.9	1,7 ± 0.6	1.1 ± 0.5	6.4 ± 4.6
≥65 (*n* = 21)	0.7 ± 0.3	14.0 ± 5.0	1.6 ± 0.4	2.8 ± 0.8	1.7 ± 0.5	2.7 ± 0.8	1.6 ± 0.5	1.1 ± 0.4	6.0 ± 3.6
Race									
Hispanic or Latinx (*n* = 53)	0.7 ± 0.4	15.3 ± 7.3	1.7 ± 0.9	2.9 ± 0.8	1.7 ± 0.5	2.8 ± 0.9	1.7 ± 0.5	1.2 ± 0.6	6.6 ± 5.4
Non-Hispanic White (*n* = 24)	0.7 ± 0.4	18.2 ± 9.3	2.0 ± 0.7	2.7 ± 1.0	1.7 ± 0.5	2.6 ± 1.0	1.6 ± 0.6	1.0 ± 0.6	10.1 ± 9.2
Other ^1^ (*n* = 6)	1.0 ± 0.7	17.3 ± 10.5	1.6 ± 1.0	3.1 ± 0.8	1.9 ± 0.6	2.9 ± 0.8	1.8 ± 0.6	1.1 ± 0.4	6.2 ± 3.8
Education									
Less than high school (*n* = 32)	0.8 ± 0.4	14.6 ± 5.5	1.7 ± 0.6	3.0 ± 0.8	1.8 ± 0.6	2.9 ± 0.8	1.7 ± 0.6	1.2 ± 0.5	5.6 ± 2.4
Completed high school (*n* = 29)	0.7 ± 0.4	18.4 ± 10.5	1.9 ± 0.6	2.7 ± 0.9	1.6 ± 0.5	2.6 ± 1.0	1.5 ± 0.5	1.1 ± 0.5	9.9 ± 9.5
Some college or more (*n* = 22)	0.6 ± 0.4	15.9 ± 7.5	1.8 ± 1.2	2.8 ± 1.0	1.8 ± 0.5	2.8 ± 1.0	1.7 ± 0.5	1.1 ± 0.8	7.3 ± 6.0
Employment									
Employed (*n* = 18)	0.8 ± 0.3	17.1 ± 9.5	2.1 ± 1.4	3.2 ± 1.2	1.9 ± 0.6	3.1 ± 1.3	1.8 ± 0.6	1.3 ± 0.9	8.0 ± 7.6
Unemployed (*n* = 28)	0.8 ± 0.5	17.0 ± 8.8	1.7 ± 0.6	2.7 ± 0.7	1.7 ± 0.4	2.6 ± 0.7	1.6 ± 0.5	1.1 ± 0.5	7.8 ± 6.1
Not seeking employment ^2^ (*n* = 37)	0.7 ± 0.3	15.4 ± 7.0	1.7 ± 0.6	2.8 ± 0.8	1.7 ± 0.5	2.7 ± 0.9	1.6 ± 0.6	1.1 ± 0.4	7.2 ± 7.0
Annual Household Income									
Less than USD 5000 (*n* = 11)	1.0 ± 0.6	19.4 ± 12.5	2.0 ± 0.9	3.1 ± 0.9	1.9 ± 0.6	3.0 ± 0.9	1.8 ± 0.6	1.1 ± 0.5	10.1 ± 11.3
USD 5001–10,000 (*n* = 24)	0.7 ± 0.4	16.0 ± 8.1	1.8 ± 1.1	2.8 ± 1.0	1.7 ± 0.5	2.7 ± 1.0	1.6 ± 0.5	1.2 ± 0.7	7.7 ± 7.0
USD 10,001–15,000 (*n* = 19)	0.7 ± 0.3	17.2 ± 8.5	1.8 ± 0.5	2.8 ± 0.7	1.7 ± 0.5	2.7 ± 0.8	1.6 ± 0.5	1.2 ± 0.5	7.5 ± 6.2
USD 15,001–30,000 (*n* = 20)	0.7 ± 0.3	13.5 ± 3.0	1.7 ± 0.7	2.8 ± 0.8	1.7 ± 0.5	2.7 ± 0.9	1.7 ± 0.5	1.0 ± 0.4	5.5 ± 2.5
More than USD 30,000 (*n* = 8)	0.7 ± 0.4	17.8 ± 8.8	1.7 ± 0.6	3.0 ± 1.3	1.8 ± 0.7	2.9 ± 1.3	1.8 ± 0.8	1.2 ± 0.8	9.2 ± 7.3
Poverty Status ^3^									
Above poverty (*n* = 29)	0.7 ± 0.4	14.8 ± 5.6	1.6 ± 0.6	2.8 ± 0.9	1.7 ± 0.5	2.7 ± 0.9	1.6 ± 0.6	1.1 ± 0.5	6.7 ± 4.5
Poverty (*n* = 32)	0.7 ± 0.3	16.8 ± 7.6	1.8 ± 0.5	2.7 ± 0.8	1.6 ± 0.5	2.6 ± 0.8	1.5 ± 0.5	1.1 ± 0.5	7.5 ± 6.2
Extreme poverty (*n* = 21)	0.8 ± 0.5	17.7 ± 11.4	2.0 ± 1.3	3.2 ± 1.0	1.9 ± 0.5	3.2 ± 1.0	1.9 ± 0.5	1.3 ± 0.7	8.8 ± 9.8
Marital Status									
Married or living with partner (*n* = 21)	0.7 ± 0.4	13.6 ± 3.4	1.8 ± 0.5	2.8 ± 0.7	1.8 ± 0.4	2.6 ± 0.8 *	1.7 ± 0.5	1.0 ± 0.4 *	5.6 ± 2.7
Widowed, divorced or separated (*n* = 21)	0.6 ± 0.3	15.1 ± 5.8	1.6 ± 0.5	2.5 ± 0.7	1.5 ± 0.3	2.4 ± 0.7	1.4 ± 0.4	0.9 ± 0.4	6.9 ± 5.3
Single or never married (*n* = 41)	0.8 ± 0.4	18.3 ± 10.2	1.9 ± 1.1	3.1 ± 1.0	1.8 ± 0.6	3.0 ± 1.0	1.7 ± 0.6	1.3 ± 0.7	8.9 ± 8.5
Last Food Pantry Visit									
Less than 7 days ago (*n* = 17)	0.9 ± 0.5	16.1 ± 8.2	1.9 ± 0.8	3.0 ± 1.1	1.8 ± 0.6	2.8 ± 1.1	1.7 ± 0.6	1.1 ± 0.6	8.3 ± 7.3
Less than 2-4 weeks ago (*n* = 28)	0.7 ± 0.3	15.7 ± 6.9	1.8 ± 0.6	2.9 ± 0.8	1.8 ± 0.6	2.7 ± 0.9	1.7 ± 0.6	1.0 ± 0.4	7.2 ± 7.2
More than 1 month ago (*n* = 34)	0.7 ± 0.4	16.7 ± 9.6	1.8 ± 1.0	2.9 ± 0.9	1.7 ± 0.5	2.9 ± 0.9	1.6 ± 0.5	1.3 ± 0.6	7.7 ± 6.6
Assistance Program Participation ^4^									
0 (*n* = 12)	0.7 ± 0.3	14.1 ± 4.0	1.7 ± 0.7	3.1 ± 0.7	1.8 ± 0.4	3.0 ± 0.8	1.7 ± 0.4	1.2 ± 0.5 *	5.9 ± 2.6 *
1 (*n* = 30)	0.8 ± 0.5	15.9 ± 7.6	1.8 ± 0.8	2.8 ± 0.8	1.8 ± 0.6	2.7 ± 1.0	1.7 ± 0.6	1.0 ± 0.5	7.6 ± 5.3
2 (*n* = 31)	0.7 ± 0.3	14.9 ± 4.8	1.7 ± 0.4	2..7 ± 0.7	1.7 ± 0.5	2.6 ± 0.8	1.6 ± 0.5	1.0 ± 0.5	5.9 ± 3.5
3 or More (*n* = 10)	0.7 ± 0.4	24.1 ± 15.4	2.4 ± 1.6	3.5 ± 1.0	1.8 ± 0.5	3.5 ± 1.1	1.8 ± 0.5	1.7 ± 0.9	15.0 ± 14.1
Food Security									
Food security (*n* = 25)	0.7 ± 0.3	17.2 ± 8.9	2.0 ± 1.2	3.1 ± 1.1	1.8 ± 0.6	3.0 ± 1.1	1.7 ± 0.6	1.3 ± 0.7	8.7 ± 7.1
Low food security (*n* = 28)	0.7 ± 0.4	14.3 ± 7.2	1.6 ± 0.6	2.9 ± 0.8	1.7 ± 0.5	2.8 ± 0.9	1.7 ± 0.5	1.2 ± 0.5	6.4 ± 5.3
Very low food security (*n* = 30)	0.8 ± 0.5	17.5 ± 8.3	1.8 ± 0.7	2.7 ± 0.7	1.7 ± 0.5	2.6 ± 0.8	1.6 ± 0.5	1.0 ± 0.4	7.8 ± 7.7

All values were presented as mean ± SD. One participant missing for intake of whole grains, dairy, and added sugars. Three participants missing for intakes of fruits and vegetable including and excluding fries. * Indicates statistical significance using a linear model (*p* < 0.05). ^1^ Non-Hispanic Black or African American, Asian or Asian American, or Multi-Racial. ^2^ Retired, full-time home-maker, or on disability. ^3^ Household income above the threshold or below the threshold [23] ^4^ Participation in SNAP, WIC, Social Security Disability Insurance, Temporary Assistance to Needy Families, Supplemental Security Income, Free or Reduced-Price School Lunch, or After School Summer Meal Programs. Based on 3-day food records of 40 participants, HEI-2015 scores were calculated and compared with the US national averages [33] (Table 4). The mean total HEI-2015 score of participants was 53.8 ± 10.5. HEI-2015 scores for whole fruits, greens and beans and seafood and plant proteins were lower than both the US adult (18–64 years) and older adult (≥65 years) population means.

In the assessment of nutrient inadequacy, intakes of vitamins C, A, E, calcium, zinc, magnesium, and folate were inadequate among at least 30% of all participants. Vitamin D intake was inadequate in 100% of participants (data not shown). There were statistically significant differences in adequacy of vitamin A, calcium, magnesium, and potassium intakes between racial groups (*p* < 0.05 for all) (Table 5). Participants below the poverty threshold had a higher zinc inadequacy than those above the poverty (*p* < 0.05). 

## 4. Discussion

This cross-sectional study sought to evaluate the association between food security, diet quality, nutrient adequacy and sociodemographic factors in mobile food pantry users in Windham County, CT. Overall, food pantry users tended to have lower quality diets than the average American. In this sample of Windham County food pantry users, most participants had low intakes of fruits, vegetables, whole grains and dairy. Correspondingly, intakes of many vitamins and minerals were lower than recommended. Due to the generally low food security and income level of participants, there was little variation in intake between the socioeconomic groups.

Fruits, vegetables, whole grains and dairy are important components of a healthy diet [26,34], but are usually under-consumed in low-income, food insecure populations [7,8,9,11]. These individuals potentially face several barriers to healthy eating, such as the high cost of fresh foods [13,14,35], a lack of nutrition education [36], and a lack of knowledge, equipment, or time to prepare healthy meals [14,37,38]. While food pantries aim to provide as many fresh foods as possible, there are often not enough fruits, vegetables and dairy to sustain users [11,39]. In the current study, mean predicted daily intakes of these key groups were also well below the recommended servings based on the Dietary Guidelines. Food pantry users in Connecticut had a higher overall HEI-2015 score (53.8 ± 10.5) compared to food pantry users in Indiana (42.3 ± 12.3) [16] and Alabama (43) [8]. This could be in part due to the foods distributed at mobile food pantries—mostly perishable, fresh produce and dairy. This conclusion is supported by previous research indicating that increasing the number of fresh foods available can improve dietary quality [37]. Many food insecure individuals must prioritize the amount of food over food quality due to their lack of financial resources, thus providing fresh foods is an extremely effective method of diet quality improvement in this population [37].

Likewise, sugar and saturated fat intake exceeded recommendations among study participants. Intakes were higher among younger participants, possibly due to high intake of processed and fast foods [40]. Low-income neighborhoods often have more fast food restaurants and convenience stores than their wealthier counterparts [14,41]. Additionally, foods of low nutritional quality (i.e., high added sugar content) tend to be more affordable than foods of high nutritional quality [14] and therefore, may contribute to the excess intake of sugar and saturated fat.

Nutrient adequacy was low in this sample, most likely due to the low intake of fruits, vegetables, whole grains and dairy. Of the 40 participants whose dietary record data were analyzed, all had insufficient intakes of vitamin D. This is rather unsurprising given that 65% of Americans consume below the EAR [42]. At least 30% of participants had intakes below the EAR for vitamins C, A, E, calcium, zinc, magnesium, and folate. These essential vitamins and minerals are found naturally in the food groups most lacking in the participants diets, and the diets of many food pantry users [7,18,39]. Race was associated with differences in micronutrient inadequacy, with Hispanic/Latinx participants having lower inadequacy of potassium and magnesium. Studies have found that Hispanic/Latinx individuals have better diet quality than their non-Hispanic White and non-Hispanic Black counterparts [20], but that acculturation reduces the diet quality of Hispanic/Latinx populations in the United States [43]. Although not statistically significant, the data indicate that those not relying on food assistance programs are more likely to be at risk for nutrient inadequacy—suggesting the importance of food assistance programs. This finding is consistent with the studies finding that both food secure and insecure participants have enhanced diet variety and quality following food pantry participation [10,16].

When associations between income levels and nutrient adequacy were assessed, there were few significant findings. This is most likely due to the fact that the majority of participants had an annual income of less than USD 15,000, which is below the required income for one adult in the area [42]. When diet quality and food pantry participation was studied in Indiana, there was a strong correlation between diet quality and food pantry visits, which was not replicated in the current study [16]. This could be explained by the small sample size and lack of economically diverse individuals.

A characterization of food pantry participants in Northeastern Connecticut has not been conducted previously. There are also few studies evaluating the diet quality of individuals using mobile food pantries, which often have more fresh fruits and vegetables than traditional pantries. Due to the sociodemographic diversity within the community, understanding the make-up of pantry participants was essential. In this study, notable patterns were identified, such as the largely Hispanic or Latinx make-up and high food insecurity rate, compared to national and state averages. There were also relatively low intakes of fruits, vegetables and whole grains. A significant percentage of participants also had intakes below the EAR for vitamins A, C, D, E, calcium, zinc, magnesium, and folate. Components of moderation, such as added sugar, SSBs, and saturated fat were consumed in excess, particularly among participants who were younger, male, or participating in 3 or more assistance programs. While HEI scores among this population were lower than national averages, they were higher than other studies among traditional food pantry users around the country [8,16]. Food security and diet quality were low in this population across all subgroups, making the differentiation of diet quality and nutrient adequacy between sociodemographic groups challenging. There were also few significant differences by assistance program use due to the high usage among participants.

The current study has several strengths. First, all surveys were administered by research staff, improving the accuracy of responses. Additionally, the use of a 3-day food record and the DSQ allows for a valid representation of the participant’s diet quality directly following the food pantry visit, as well as an idea of their overall eating habits [28]. Some limitations of this study are the small sample size and lack of economic diversity among recruited participants. Only 83 participants contributed to the DSQ data analysis, and 40 were included for nutrient adequacy determinations. This small sample size may be a significant source of error when assessing specific nutrient intakes, but the pattern of dietary disparities in the current study is aligned with previous research in populations of a similar socioeconomic background [8,16,18]. The current study is still a useful tool in determining certain key areas of concern regarding the diet quality and nutrient adequacy of mobile food pantry users.

Promotion of increased fruit, vegetable, dairy and whole grain intake could improve diet quality and nutrient adequacy in this population. Most participants had low intakes of key nutrients, many of which are found in these foods. Due to the high prevalence of severely low-income individuals, interventions should be focused on low cost, sustainable improvements that can be adopted to improve diet quality. One possible solution is the promotion of canned and frozen fruits and vegetables to supplement food pantry parcels due to their cost effectiveness and high nutritional value. Teaching participants how to prepare the food they have available in a healthy way should also be a priority, as this has been shown to further improve diet quality [43]. The cultural competence of interventions should also be considered due to the high prevalence of Hispanic and Latinx individuals. By heeding these findings, future interventions can make a lasting impact on the nutrition and health of mobile food pantry users in Windham County, CT.

## Figures and Tables

**Table 1:** General characteristics of study participants recruited from mobile food pantries in Windham County, CT (*n* = 83).

Characteristics	*n*	%
Sex		
Male	16	19.3
Female	67	80.7
Age (years)		
19–30	8	9.6
31–44	13	15.7
45–64	41	49.4
≥65	21	25.3
Race		
Hispanic or Latinx	53	63.9
Non-Hispanic White	24	28.9
Other ^1^	6	7.2
Education		
Less than high school	32	38.6
Completed high school	29	34.9
Some college or more	22	26.5
Employment		
Employed	18	21.7
Unemployed	28	33.7
Not seeking employment ^2^	37	44.6
Annual Household Income		
Less than USD 5000	11	13.4
USD 5001–10,000	24	29.3
USD 10,001–15,000	19	23.2
USD 15,001–30,000	20	24.4
More than USD 30,000	8	9.8
Poverty Status ^3^		
Above poverty	29	35.4
Poverty	32	39.0
Extreme poverty	21	25.6
Food Security		
Food secure	25	30.1
Low food security	28	33.7
Very low food security	30	36.1
Marital Status		
Married or living with partner	21	25.3
Widowed, divorced or separated	21	25.3
Single or never married	41	49.4
Last Food Pantry Visit		
Less than 7 days ago	17	21.5
Less than 2–4 weeks ago	28	35.4
More than 1 month ago	34	43.0
Assistance Program Participation ^4^		
0	12	14.5
1	30	36.1
2	31	37.4
3 or More	10	12.1

^1^ Non-Hispanic Black or African American, Asian or Asian American, or Multi-Racial. ^2^ Retired, full-time home-maker, or on disability. ^3^ Household income above the threshold or below the threshold [23]. ^4^ Participation in SNAP, WIC, Social Security Disability Insurance, Temporary Assistance to Needy Families, Supplemental Security Income, Free or Reduced-Price School Lunch, or After School Summer Meal Programs.

**Table 2:** USDA Dietary Guidelines for Adults by Food Group.

Food Group	USDA Dietary Guidelines for Adults ^1,2^
Whole grain (oz)	6
Dairy (cup)	3
Fruits and vegetables including legumes and French fries (cup)	4.5
Vegetables including legumes and including French fries (cup)	2.5
Fruits and vegetables including legumes and excluding French fries(cup)	4.5
Vegetables including legumes and excluding French fries (cup)	2.5
Fruits (cup)	2
Added sugars (tsp) ^3^	12
Sugar-sweetened beverages (tsp sugar) ^3^	12

^1^ USDA Dietary Guidelines [26]. ^2^ Based on a 2000 calorie diet.^3^ Components of moderation [32].

**Table 4:** Comparison of Healthy Eating Index 2015 scores between mobile food pantry users who completed 3-day food records (*n* = 40) and the US national average.

Components (Maximum Points) ^1^	Study Participants	US Population ^2^	
		18–64 y	≥65 y
	Mean ± SD	Mean	Mean
Total Score (100)	53.8 ± 10.5	58.0	65.5
Total Fruits (5)	2.7 ± 1.7	2.4	3.7
Whole Fruits (5)	2.3 ± 1.7	3.5	5.0
Total Vegetables (5)	3.5 ± 1.2	3.3	3.9
Greens and Beans (5)	2.1 ± 1.8	3.2	3.3
Whole Grains (10)	2.9 ± 2.8	2.5	4.0
Dairy (10)	4.4 ± 2.8	5.9	5.9
Total Protein Foods (5)	4.4 ± 2.1	5.0	5.0
Seafood and Plant Proteins (5)	2.1 ± 1.8	5.0	5.0
Fatty Acids (10)	4.9 ± 2.4	4.6	5.0
Refined Grains ^3^ (10)	6.0 ± 2.4	6.3	7.6
Sodium ^3^ (10)	3.7 ± 2.2	3.9	4.0
Added Sugar ^3^ (10)	8.7 ± 2.2	6.4	7.5
Saturated Fat ^3^ (10)	6.2 ± 2.3	6.0	5.7

^1^ Maximum possible score is indicated for each dietary component, with a higher score indicating greater adherence to the Dietary Guidelines 2015–2020. ^2^ Average HEI-2015 scores for US Adults according to the USDA [33]. ^3^ For refined grains, sodium, added sugar and saturated fat, a higher score reflects more moderate consumption. For all other components, a higher score reflects higher intake.

**Table 5:** Nutrient inadequacy based on 3-day dietary records of mobile food pantry users by sociodemographic factors (*n* = 40).

Sociodemographic Characteristics	% < AMDR or > AMDR ^3^				% < EAR ^4^								% > AI ^5^		
	n	Fat	Protein	Carb	Vit C	Vit A	Vit E	Folate	Ca	Fe	Mg	Zn	Fiber	K	Na
Sex															
Male	5	40.0	0.0	40.0	60.0	80.0	80.0	20.0	80.0	0.0	80.0	60.0	20.0	20.0	100.0
Female	35	37.1	2.9	40.0	28.6	48.6	100.0	28.6	77.1	2.9	68.6	25.7	22.9	31.4	97.1
Age															
19–50	10	40.0	0.0	30.0	40.0	40.0	100.0	10.0	70.0	10.0	70.0	10.0	10.0	30.0	100.0
51–70	23	34.8	4.4	43.5	30.4	56.5	95.7	39.1	78.3	0.0	69.6	34.8	30.4	30.4	100.0
70+	7	42.9	0.0	42.9	28.6	57.1	100.0	14.3	85.7	0.0	71.4	42.9	14.3	28.6	85.7
Race															
Hispanic or Latinx	25	32.0	4.0	36.0	28.0	68.0 *	96.0	20.0	92.0 *	0.0	72.0 *	32.0	24.0	28.0 *	96.0
Non-Hispanic White	12	58.3	0.0	58.3	50.0	33.3	100.0	50.0	66.7	8.3	83.3	33.3	16.7	16.7	100.0
Other ^1^	3	0.0	0.0	0.0	0.0	0.0	100.0	0.0	0.0	0.0	0.0	0.0	33.3	100.0	100.0
Annual Household Income															
Less than USD 10,000	13	38.5	0.0	23.1	30.8	69.2 *	92.3	38.5	76.9 *	7.7	76.9	38.5 *	23.1	23.1	92.3
USD 10,001–15,000	12	41.7	0.0	66.7	50.0	75.0	100.0	25.0	100.0	0.0	83.3	50.0	16.7	16.7	100.0
More than USD 15,000	14	28.6	7.1	28.6	21.4	21.4	100.0	21.4	57.1	0.0	51.1	7.1	28.6	42.9	100.0
Poverty Status ^2^															
Above threshold	18	27.8	5.6	33.3	22.2	38.9	100.0	22.2	66.7	0.0	61.1	11.1 *	27.8	38.9	100.0
Below threshold	21	42.9	0.0	42.9	42.9	66.7	95.2	33.3	85.7	4.8	81.0	47.6	19.1	19.1	95.2
Marital Status															
Married or living with partner	10	50.0	0.0	60.0	50.0	50.0	100.0	40.0	90.0	10.0	90.0	40.0	10.0	10.0	100.0
Widowed, divorced or separated	12	33.3	8.3	25.0	25.0	58.3	100.0	25.0	91.7	0.0	83.3	33.3	8.3	16.7	91.7
Single or never married	18	33.3	0.0	38.9	27.8	50.0	94.4	22.2	61.1	0.0	50.0	22.2	38.9	50.0	100.0

* indicates statistical significance using Chi-Square test (*p* < 0.05). ^1^ Non-Hispanic Black or African American, Asian or Asian American, or Multi-Racial. ^2^ Household income above the threshold or below the threshold [23]. ^3^ Acceptable Macronutrient Distribution Range. ^4^ Estimated Average Requirement. ^5^ Adequate Intake.

## Data Availability

All relevant data for this study is included in the manuscript.
